# Supplementary material for: Climate, inter-serotype competition and arboviral interactions shape dengue dynamics in Thailand
Source: Commun Biol. 2025 Apr 11;8:601. doi: 10.1038/s42003-025-07999-9 (PMC11992266; doi:10.1038/s42003-025-07999-9)
Supplement: Supplementary file 1 — Supplementary Information [file 42003_2025_7999_MOESM1_ESM.pdf]

Supplementary Material

Title: Climate, inter-serotype competition and arboviral interactions shape dengue dynamics in Thailand

Lester J. Perez[1,2], Julie Yamaguchi[1,2], Sonja Weiss[1,2], Christiane Carlos[1,2], Todd V. Meyer[1,2], Mary A. Rodgers[1,2], Pakpoom Phoompoung[3], Yupin Suputtamongkol[3], Gavin A. Cloherty[1,2], Michael G. Berg[1,2].

[1]Infectious Disease Research, Abbott Diagnostics Division, Abbott Laboratories, Abbott Park, Illinois, USA.
[2]Abbott Pandemic Defense Coalition (APDC), Abbott Park, Illinois, USA.
[3]Faculty of Medicine, Siriraj Hospital Mahidol University, Bangkok, Thailand.

*to whom correspondence should be addressed: Lester J. Perez, lester.perez@abbott.com

*to whom correspondence should be addressed: Lester J. Perez, lester.perez@abbott.com

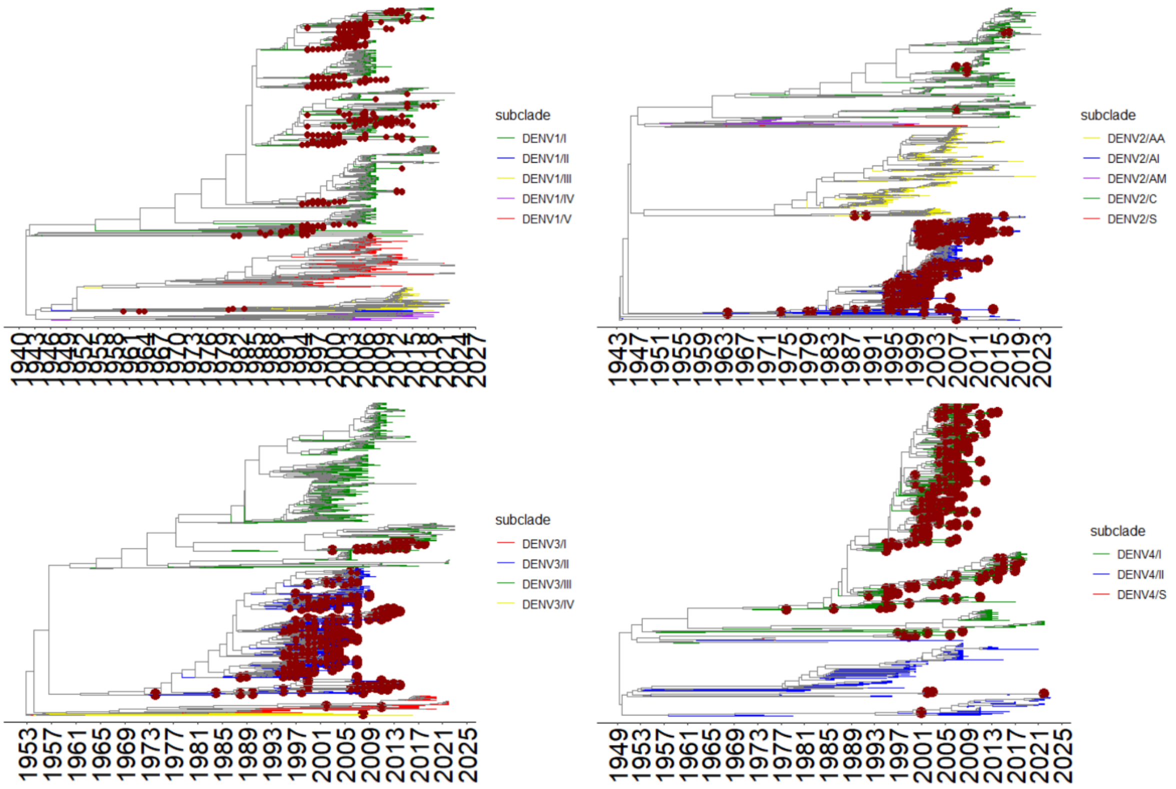

Figure S1. Time-stamped phylogenies represented by MCC-tree for all four DENV serotypes. Sequences from Thailand are highlighting with red tips. In these cases, all genotypes within each serotype were denoted with different colors.

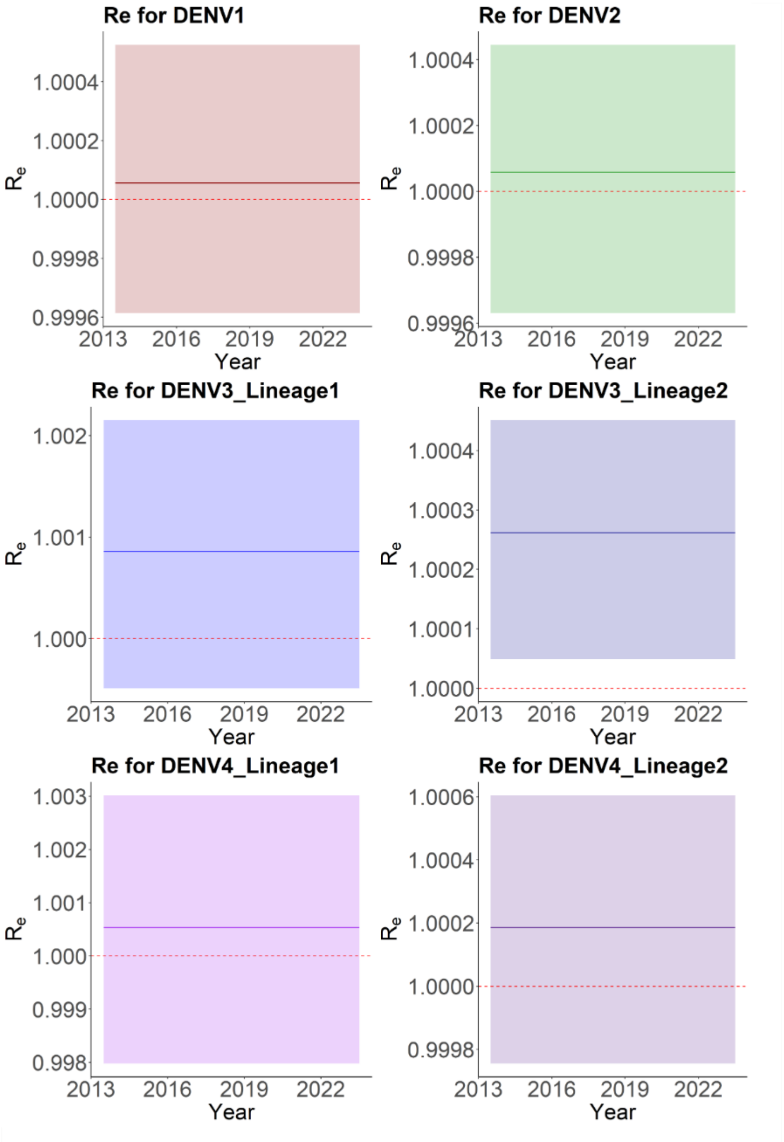

Figure S2. Assessment of Effective Reproductive Rates (Re) for each DENV serotype and lineage using the Birth-Death Skyline (BdSk). Evaluation of Re for each monophyletic lineage circulating in Thailand (as endemic circulation), each lineage and serotype are distinctly marked to illustrate differential transmission potentials over time.

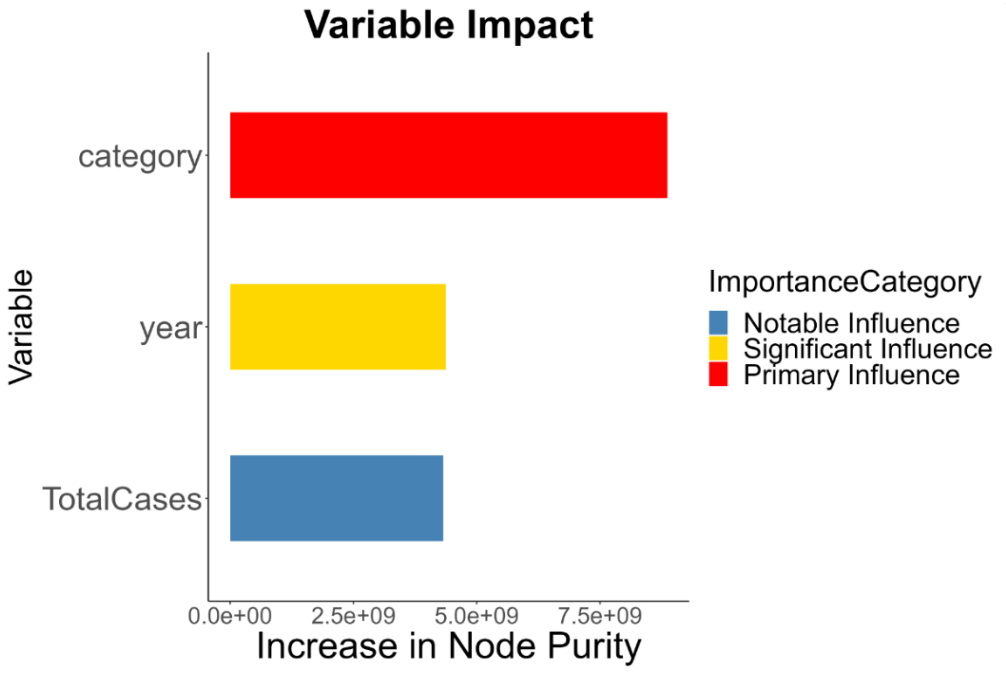

Figure S3. Impact assessment of contributory variables using random forest analysis. The application of a random forest model to evaluate the force of infection (FOI) for dengue virus (DENV) in Thailand from 2014 to 2024. Variables included in the model span clinical manifestations of the disease such as dengue fever, hemorrhagic dengue fever, and shock due to dengue fever, along with annual deaths. It specifically highlights the influence of concurrent Chikungunya virus (CHIKV) cases on dengue virus (DENV) dynamics.

Table S1. Estimated substitution rates for the DENV endemic main clades

Endemic Lineage	Evolutionary rates [HPD95%] (substitution/site/year)
DENV1	7.98×10^{-4} [7.56×10^{-4} - 8.41×10^{-4}]
DENV2	7.67×10^{-4} [7.21×10^{-4} - 8.12×10^{-4}]
DENV3 L1	9.01×10^{-4} [7.9×10^{-4} - 1.13×10^{-3}]
DENV3 L2	2.40×10^{-4} [2.03×10^{-4} - 2.6×10^{-4}]
DENV4 L1	4.80×10^{-4} [2.03×10^{-4} - 8.3×10^{-4}]
DENV4 L2	6.62×10^{-3} [6.07×10^{-4} - 7.21×10^{-4}]
