# Supplementary material for: Climate, inter-serotype competition and arboviral interactions shape dengue dynamics in Thailand
Source: Commun Biol. 2025 Apr 11;8:601. doi: 10.1038/s42003-025-07999-9 (PMC11992266; doi:10.1038/s42003-025-07999-9)
Supplement: Supplementary file 2 — Description of Additional Supplementary Files [file 42003_2025_7999_MOESM2_ESM.pdf]

Description of Additional Supplementary Files

File Name: Supplementary Data 1
Description: aBSREL results DENV1.

File Name: Supplementary Data 2
Description: FUBAR DENV1.

File Name: Supplementary Data 3
Description: FUBAR DENV2.

File Name: Supplementary Data 4
Description: abSREL results DENV2.

File Name: Supplementary Data 5
Description: MEME DENV1.

File Name: Supplementary Data 6
Description: FUBAR DENV3.

File Name: Supplementary Data 7
Description: abSREL DENV3.

File Name: Supplementary Data 8
Description: MEME DENV2.

File Name: Supplementary Data 9
Description: FUBAR DENV4.

File Name: Supplementary Data 10
Description: abSREL DENV4.

File Name: Supplementary Data 11
Description: MEME DENV3.

File Name: Supplementary Data 12
Description: MEME DENV4.

File Name: Supplementary Data 13
Description: Function of residues identified under positive selection.

File Name: Supplementary Video 1
Description: Time Series of Index P in Thailand.

File Name: Supplementary Video 2
Description: Time Series of dengue fever cases in Thailand.

File Name: Supplementary Video 3
Description: Time of chikungunya cases in Thailand.
