# Supplementary material for: Climate, inter-serotype competition and arboviral interactions shape dengue dynamics in Thailand
Source: Commun Biol. 2025 Apr 11;8:601. doi: 10.1038/s42003-025-07999-9 (PMC11992266; doi:10.1038/s42003-025-07999-9)
Supplement: Supplementary file 19 — Reporting summary [file 42003_2025_7999_MOESM19_ESM.pdf]

nature portfolio

Corresponding author(s): Lester J. Perez

Last updated by author(s): Oct 3, 2024

Reporting Summary

Nature Portfolio wishes to improve the reproducibility of the work that we publish. This form provides structure for consistency and transparency in reporting. For further information on Nature Portfolio policies, see our Editorial Policies and the Editorial Policy Checklist.

Statistics

For all statistical analyses, confirm that the following items are present in the figure legend, table legend, main text, or Methods section.

n/a	Confirmed	
☐	☒	The exact sample size (*n*) for each experimental group/condition, given as a discrete number and unit of measurement
☒	☐	A statement on whether measurements were taken from distinct samples or whether the same sample was measured repeatedly
☒	☐	The statistical test(s) used AND whether they are one- or two-sided<br>*Only common tests should be described solely by name; describe more complex techniques in the Methods section.*
☒	☐	A description of all covariates tested
☒	☐	A description of any assumptions or corrections, such as tests of normality and adjustment for multiple comparisons
☒	☐	A full description of the statistical parameters including central tendency (e.g. means) or other basic estimates (e.g. regression coefficient) AND variation (e.g. standard deviation) or associated estimates of uncertainty (e.g. confidence intervals)
☐	☒	For null hypothesis testing, the test statistic (e.g. *F*, *t*, *r*) with confidence intervals, effect sizes, degrees of freedom and *P* value noted<br>*Give P values as exact values whenever suitable.*
☐	☒	For Bayesian analysis, information on the choice of priors and Markov chain Monte Carlo settings
☒	☐	For hierarchical and complex designs, identification of the appropriate level for tests and full reporting of outcomes
☒	☐	Estimates of effect sizes (e.g. Cohen's *d*, Pearson's *r*), indicating how they were calculated

Our web collection on statistics for biologists contains articles on many of the points above.

Software and code

Policy information about availability of computer code

Data collection	Leftover plasma samples were collected during 2014-2023 from patients presenting with acute febrile illness and stored at -80ºC. Individuals testing positive by Dengue qPCR (n=186) were sequenced as described below.
Data analysis	Sequencing data were processed using Qiagen CLC Genomics Workbench, where reads were mapped to reference sequences of all four dengue virus (DENV) serotypes to generate consensus sequences. Sequences were aligned using MAFFT with the "localpair" option. Maximum likelihood phylogenetic trees were constructed using IQ-TREE2. Temporal phylogenies were generated using BEAST 1.10.5 with an uncorrelated relaxed lognormal clock and a Gaussian Markov random field model to estimate evolutionary rates and divergence times. Eight independent Markov Chain Monte Carlo runs were executed, and convergence was confirmed with effective sample sizes exceeding 200 in Tracer v1.7. The effective reproduction numbers (Re) of the DENV lineages were estimated using the Birth-Death Skyline (BDSKY) model in BEAST 2.7.6. Discrete phylogeographic analyses were conducted to investigate the geographical spread and origins of DENV serotypes in Thailand.. Markov jump counts were estimated to quantify state transitions between geographical regions, and results were visualized using the R package circlize.

For manuscripts utilizing custom algorithms or software that are central to the research but not yet described in published literature, software must be made available to editors and reviewers. We strongly encourage code deposition in a community repository (e.g. GitHub). See the Nature Portfolio guidelines for submitting code & software for further information.

Data

Policy information about availability of data

All manuscripts must include a data availability statement. This statement should provide the following information, where applicable:

- Accession codes, unique identifiers, or web links for publicly available datasets
- A description of any restrictions on data availability
- For clinical datasets or third party data, please ensure that the statement adheres to our policy

All sequences and their associated metadata used in this study are available on Zenodo (https://doi.org/10.5281/zenodo.13883087).
Epidemiological data were obtained from the Thailand Ministry of Public Health's surveillance portal (http://doe.moph.go.th/surdata/disease.php).
Climatic data were sourced from publicly available satellite data provided by Copernicus.eu, specifically the "ERA5-Land monthly averaged data from 2014 to 2023" dataset (https://cds.climate.copernicus.eu/cdsapp#!/dataset/reanalysis-era5-land-monthly-means?tab=overview), which we used to parameterize our models.
Genbank Accession number from the sequences generated in this study are available in Data/Table at https://github.com/LesterJP/DENV-Thailand.

Research involving human participants, their data, or biological material

Policy information about studies with human participants or human data. See also policy information about sex, gender (identity/presentation), and sexual orientation and race, ethnicity and racism.

Reporting on sex and gender	Patients presenting with acute febrile illness were recruited to study irrespective of sex or gender. From those patients in which Dengue sequences were obtained and for which this information was recorded, approximately 46% were female and 54% were male.
Reporting on race, ethnicity, or other socially relevant groupings	Patients were all of Asian descent and citizens of Thailand. Hospital and recruitment sites were in the cities of Bangkok and Korat
Population characteristics	Patients ranged in age from 15-72 years old. The median age was 24. Those given a diagnosis or dengue fever or dengue hemorrhagic fever frequently had corroborating positive dengue real time RT-PCR results with viral loads. Sequencing of plasma by NGS further confirmed dengue.
Recruitment	In-patients at Siraraj Hospital of Mahidol University in Bangkok, Thailand presenting with acute febrile illness from 2014-2023 were retrospectively analyzed by metagenomic and target enriched NGS. Those with a tentative or confirmed (RT-qPCR+) diagnosis of dengue fever and also positive by NGS were characterized phylogenetically in this study. Additional patients with only dengue-positive NGS results were also included. For those enrolled prospectively in the larger study after 2022, case definitions for syndromes included unexplained fever, hepatitis, respiratory illness, rash, encephalitis, meningitis, gastroenteritis and acute diarrhea were followed for patient selection. Once again, only those yielding dengue genomes were included in this analysis.
Ethics oversight	This study was approved by Siriraj Institutional Review Board committee in Thailand (COA Si.391/2021). Individuals provided written informed consent.

Note that full information on the approval of the study protocol must also be provided in the manuscript.

Field-specific reporting

Please select the one below that is the best fit for your research. If you are not sure, read the appropriate sections before making your selection.

☒ Life sciences ☐ Behavioural & social sciences ☐ Ecological, evolutionary & environmental sciences

For a reference copy of the document with all sections, see nature.com/documents/nr-reporting-summary-flat.pdf

Life sciences study design

All studies must disclose on these points even when the disclosure is negative.

Sample size	We analyzed a total of 186 individual samples that tested positive for dengue virus (DENV) via real-time RT-qPCR. These positive cases were identified from a cohort of 2 000 patients presenting with Acute Febrile Illness (AFI) collected between 2014 and 2023. The DENV-positive samples represent approximately 9.3% of the total cohort. By including all available positive samples from this longitudinal study, we aimed to capture a comprehensive picture of the evolution and transmission dynamics of DENV in Thailand over the nine-year period. The sample size was inherently determined by the number of positive detections within the cohort, and although limited by the prevalence of DENV among AFI cases, it was sufficient to provide significant insights into the viral genetic diversity and epidemiological trends in the region.
Data exclusions	No data were excluded from this study, as our objective was to collect as many new dengue virus (DENV) genomes as possible
Replication	Sequencing and real-time RT-qPCR experiments were not replicated due to the limited volume of samples available in the study.

Randomization	Randomization was not applied in this study because we included all available positive samples from a longitudinal dataset spanning from 2012 to 2023. Our objective was to obtain a comprehensive understanding of the evolution and transmission dynamics of dengue virus (DENV) in Thailand over this period. By analyzing all positive cases, we aimed to capture the full genetic diversity and temporal changes of DENV circulating in the region.Using the entire dataset allowed us to identify emerging lineages, track the spread of different serotypes, and assess evolutionary rates without the biases that might be introduced by selecting a random subset of samples. Randomization in this context could have led to the exclusion of significant variants or transmission events, thereby limiting the study's ability to accurately reflect the epidemiological landscape of DENV in Thailand.
Blinding	Blinding was not applicable in this study because our aim was to analyze the transmission dynamics of dengue virus (DENV) in Thailand and its role in the global epidemic. The research involved the analysis of publicly available epidemiological and genomic data, without experimental interventions or assessments where blinding would be relevant.

Reporting for specific materials, systems and methods

We require information from authors about some types of materials, experimental systems and methods used in many studies. Here, indicate whether each material, system or method listed is relevant to your study. If you are not sure if a list item applies to your research, read the appropriate section before selecting a response.

Materials & experimental systems

n/a	Involved in the study	
☒	☐	Antibodies
☒	☐	Eukaryotic cell lines
☒	☐	Palaeontology and archaeology
☒	☐	Animals and other organisms
☒	☐	Clinical data
☒	☐	Dual use research of concern
☒	☐	Plants

Methods

n/a	Involved in the study	
☒	☐	ChIP-seq
☒	☐	Flow cytometry
☒	☐	MRI-based neuroimaging

Plants

Seed stocks	*Report on the source of all seed stocks or other plant material used. If applicable, state the seed stock centre and catalogue number. If plant specimens were collected from the field, describe the collection location, date and sampling procedures.*
Novel plant genotypes	*Describe the methods by which all novel plant genotypes were produced. This includes those generated by transgenic approaches, gene editing, chemical/radiation-based mutagenesis and hybridization. For transgenic lines, describe the transformation method, the number of independent lines analyzed and the generation upon which experiments were performed. For gene-edited lines, describe the editor used, the endogenous sequence targeted for editing, the targeting guide RNA sequence (if applicable) and how the editor was applied.*
Authentication	*Describe any authentication procedures for each seed stock used or novel genotype generated. Describe any experiments used to assess the effect of a mutation and, where applicable, how potential secondary effects (e.g. second site T-DNA insertions, mosiacism, off-target gene editing) were examined.*
